# Polymorphic markers for identification of parasite population in *Plasmodium malariae*

**DOI:** 10.1186/s12936-020-3122-2

**Published:** 2020-01-28

**Authors:** Vivek Bhakta Mathema, Supatchara Nakeesathit, Watcharee Pagornrat, Frank Smithuis, Nicholas J. White, Arjen M. Dondorp, Mallika Imwong

**Affiliations:** 1 Department of Molecular Tropical Medicine and Genetics, Faculty of Tropical Medicine, Mahidol University, Bangkok, 10400 Thailand; 2 Mahidol–Oxford Tropical Medicine Research Unit, Faculty of Tropical Medicine, Mahidol University, Bangkok, Thailand; 3 Medical Action Myanmar, Yangon, Myanmar; 4 Centre for Tropical Medicine and Global Health, Nuffield Department of Medicine, University of Oxford, Oxford, UK

**Keywords:** Genotyping, INDEL, Nucleotide diversity, Tandem repeats

## Abstract

**Background:**

Molecular genotyping in *Plasmodium* serves many aims including providing tools for studying parasite population genetics and distinguishing recrudescence from reinfection. Microsatellite typing, insertion-deletion (INDEL) and single nucleotide polymorphisms is used for genotyping, but only limited information is available for *Plasmodium malariae*, an important human malaria species. This study aimed to provide a set of genetic markers to facilitate the study of *P. malariae* population genetics.

**Methods:**

Markers for microsatellite genotyping and *pmmsp1* gene polymorphisms were developed and validated in symptomatic *P. malariae* field isolates from Myanmar (N = 37). Fragment analysis was used to determine allele sizes at each locus to calculate multiplicity of infections (MOI), linkage disequilibrium, heterozygosity and construct dendrograms. Nucleotide diversity (π), number of haplotypes, and genetic diversity (*H*_*d*_) were assessed and a phylogenetic tree was constructed. Genome-wide microsatellite maps with annotated regions of newly identified markers were constructed.

**Results:**

Six microsatellite markers were developed and tested in 37 *P. malariae* isolates which showed sufficient heterozygosity (0.530–0.922), and absence of linkage disequilibrium (*I*_*A*_^*S*^=0.03, *p value *>* 0.05*) (N = 37). In addition, a tandem repeat (VNTR)-based *pmmsp1* INDEL polymorphisms marker was developed and assessed in 27 *P. malariae* isolates showing a nucleotide diversity of 0.0976, haplotype gene diversity of 0.698 and identified 14 unique variants. The size of VNTR consensus repeat unit adopted as allele was 27 base pairs. The markers Pm12_426 and *pmmsp1* showed greatest diversity with heterozygosity scores of 0.920 and 0.835, respectively. Using six microsatellites markers, the likelihood that any two parasite strains would have the same microsatellite genotypes was 8.46 × 10^−4^ and was further reduced to 1.66 × 10^−4^ when *pmmsp1* polymorphisms were included.

**Conclusions:**

Six novel microsatellites genotyping markers and a set of *pmmsp1* VNTR-based INDEL polymorphisms markers for *P. malariae* were developed and validated. Each marker could be independently or in combination employed to access genotyping of the parasite. The newly developed markers may serve as a useful tool for investigating parasite diversity, population genetics, molecular epidemiology and for distinguishing recrudescence from reinfection in drug efficacy studies.

## Background

Over the past decade, the world has experienced significant reduction in global falciparum malaria burden, but this decline was less prominent for the other human *Plasmodium* species [1]. *Plasmodium malariae* is endemic throughout parts of South America, Africa, Asia, and the Western Pacific [2–4]. It has been argued that malaria elimination programmes largely focused on *Plasmodium falciparum* and *Plasmodium vivax* may have undermined *P. malariae* and *Plasmodium ovale* endemicity [5]. *Plasmodium malariae* deploys a different transmission strategy, with a large parasite reservoir in asymptomatic carriers with low parasitaemia. These chronic *P. malariae* infections can occasionally cause anaemia and nephrotic syndrome [4, 6–8]. Use of molecular techniques such as qPCR has enabled more sensitive detection of parasite carriage compared to microscopy [3, 9]. Previous studies conducted in Cameroon [10] and Equatorial Guinea [9] using qPCR based species identification revealed that *P. falciparum* was the dominating species responsible for over 80.0% of malaria positive cases, followed by *P. malariae* (≥ 12.0%). In the Colombian Amazon region, the *P. malariae* infections were reported as high as 43.2% [11]. Nevertheless, *P. malariae* is understudied compared to the other human *Plasmodium* species [12], although there are recently increased efforts to describe the epidemiology of *P. malariae* [13–17]. However, important molecular tools for this are currently lacking, which will facilitate the description of the course of natural infections, multiplicity of infection (MOI) and anti-malarial drug resistance [18, 19]. In clinical trials on drug efficacy, genetic markers are important to distinguish between recrudescence and reinfection [20, 21].

Microsatellite markers remain an important technique in population genetics because of its high level of polymorphisms and fidelity to discriminate variants within parasite population [13, 14, 22]. Microsatellites are short tandem repeats of nucleotides usually consisting of 1 to 10 base pair (bp) unit motifs [23]. These are abundant in non-coding regions of the genome, which are generally not under selection pressure, and their molecular origin and evolution result from improper alignment, mispairing and strand-slippage events [22, 24, 25]. Heterozygosity refers to observation of two different alleles at a locus which forms fundamental bases for investigation of genetic variation in population. Since blood-stage *Plasmodium* is haploid, a single-genotype infection is expected to have single allele at each location while a multiple-genotype infection is expected to carry multiple alleles [14, 20]. Defining potential microsatellite markers requires assessment of their polymorphisms, and evaluation of heterozygosity and linkage disequilibrium [20, 26]. High precision fragment analyzers based on capillarity electrophoresis have a resolution of 1–2 bp. This technology in combination with analysis software like GeneScan™ provides a powerful tool for analyses of microsatellite markers. Few adequate microsatellite markers have been identified for *P. malariae* compared to *P. falciparum* the [21, 27–29] and *P. vivax* [20, 30–32]. The previously identified few *P. malariae* genotyping markers [13, 14] showed promising potential and could identify up to 10 distinct alleles for a single locus.

**Fig. 1:**
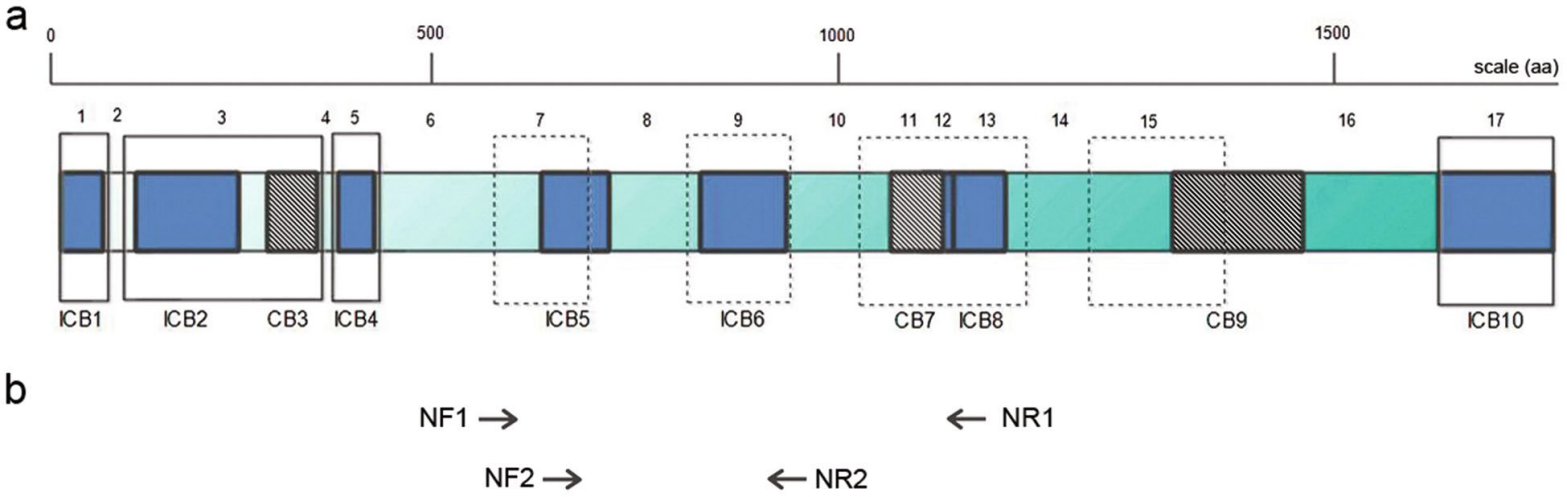
Representative block diagram of merozoite surface protein 1 [40]. **a** The block diagram for *msp1* gene is based on conserved amino acid sequences in *Plasmodium vivax, P. falciparum* and *Plasmodium yoelii* (inner block). The outer solid and dotted hollow blocks represents conserved and semi-conserved regions based on *P. falciparum* sequence. The solid block represents ICB with more than 48% identical sequences among three species. The hatched block represents more than 50% identical (conserved) sequences between *P. falciparum* and *P. vivax.* Open regions represents less than 45% identity among three species. ICB, interspecies conserved block; CB, Conserved block. **b** Schematic representation of the primary (NF1, NR1) and secondary (NF2, NR2) oligonucleotide primers used to sequence partial region of the *pmmsp1* gene

Recent developments in high-throughput sequencing technology has enabled Insertion-deletions (INDEL)-based gene polymorphisms to construct high-resolution genetic maps and evaluate parasite population structures [33]. The approach has been used for investigating polymorphisms in genes encoding gametocyte antigen, duffy binding protein and merozoite surface proteins (*msp*) [34–36]. Previous studies in *P. vivax* and *P. falciparum* have utilized gene polymorphisms in *msp* and circumsporozoite protein to study the parasite diversity [37–39]. The *msp* genes are part of a larger family that encodes the major surface antigens for invasive forms of *Plasmodium* during erythrocytic stages [40–42]. Functions for some of these proteins have described for *P. vivax* [43] and *P. falciparum* [44, 45]. Compared to microsatellite-based genotyping, the utility of surface protein gene-based markers are often limited due to reduced polymorphisms as a result of selective pressure by the host immune system [46, 47], the members of the *msp* family have been widely used as genotyping markers [36, 48]. The variable number of tandem repeats (VNTR)-related INDEL polymorphisms contributes to *msp* gene diversity [40, 48, 49]. The *msp* genes consists of exons as amino acid coding blocks interspersed between their conserved and semi-conserved blocks (Fig. 1). Sequences within these semi-conserved regions can be highly variable and thus are potential genetic markers. In this context, the performance of *pfmsp1, pfmsp2, pvmsp1* and *pvmsp3α* genes to discriminate between recrudescence and reinfection has been studied [50, 51]. The homologue gene *P. malariae* merozoite surface protein 1 (*pmmsp1*) is equally polymorphic and a candidate for parasite genotyping [52, 53]. In particular, high allelic frequency and genetic diversity in *msp1* were observed for symptomatic *P. falciparum* malaria patients in Africa [54], Thailand [38] and Burkina-Faso [55]. Apart from a few previous studies reported with limited samples for *P. malariae* infections in human [56] and monkey [57, 58], only sparse information is available on *pmmsp1* INDEL gene polymorphisms. The present study developed and validated six microsatellite genotyping markers as well as markers for *pmmsp1* polymorphisms, which can serve as a tool for epidemiological and other studies.

## Methods

### Study site, DNA sampling and reference sequences

*Plasmodium malariae* samples from symptomatic patients were collected in 2008 in Rakhine state of Myanmar (N = 37) as part of a larger study to compare the effectiveness of five artemisinin-based combination therapy regimen [59]. Parasite DNA was extracted using QIAamp DNA mini kit (Qiagen, Germany). All samples were confirmed as *P. malariae* using 18 small-subunit ribosomal RNA (18S rRNA)-based PCR [60] following the Standards for the Reporting of Diagnostic Accuracy (STARD) [61–63]. Ethical approval for the study was obtained from the ethical review board of the Faculty of Tropical Medicine, Mahidol University. Reference sequences for whole genomes of *P. malariae* UG01, *P. falciparum* 3D7*, P. vivax* SAL-1, *P. ovale curtisi* GH01 and *P. knowlesi* STRAIN-H were downloaded from PlasmoDB [64] repository (http://plasmodb.org/common/downloads/release-36/). The reference sequence for *pmmsp1* gene (Accession no. FJ824669) was obtained from NCBI nucleotide database.

### Development of genotyping markers

Identification of genome-wide perfect and imperfect microsatellites was performed using Phobos version 3.3.11 [65, 66]. The detection criteria for tandem repeat was restricted to evaluation of perfect and imperfect repeats with unit motifs of 1–10 bp having a minimum threshold repeat number of 14, 7, 5, 4, 4, 4, 4, 4, 4 and 4 for mono-, di-, tri-, tetra-, penta-, hexa-, hepta-, octa-, nonan-, and deca-nucleotide microsatellites, respectively. The genome wide distribution of microsatellites in reference sequences were summarized (Additional file 1: Table S1).

Sequences of six potential microsatellite genotyping markers together with 150 bp flanking sequences were extracted and primers were designed (Table 1) using BatchPrimer3 version 1.0 (https://probes.pw.usda.gov/cgi-bin/batchprimer3/batchprimer3.cgi). The selection criteria of primers were set as: (i) PCR product should be unique to the target flanking sequence containing the microsatellite; (ii) The variation in length of PCR product should be result of deviation in microsatellite length; (iii) Microsatellites with higher copy numbers, followed by manual inspection of each primer products to avoid selection of primers that could potentially associate with experimentally verified protein coding regions of the parasite or any interspecies cross reactivity; (iv) Preference is given to primers located at physically different chromosomes with no linkage disequilibrium. Likewise, during validation steps the factors such as ease of amplification, no starter peaks and absence of non-specific bands were also considered for selection of microsatellite markers. To investigate polymorphisms in *pmmsp1* gene, the partial regions of *pmmsp1* genes were amplified using semi-nested PCR with specifically designed primer sets (Table 2). Allelic frequencies were determined based on length of consensus sequences for variable number of tandem repeats (VNTR) in semi-conserved regions interspersed between coding blocks (Fig. 1). Only the well-amplified VNTR in majority of samples with copy numbers greater than 2.0 were considered as alleles for INDEL polymorphisms analysis.

**Table 1:** List of candidate genotyping microsatellite markers for *Plasmodium malariae* UG01

S. no.	Locus^a^	Primer	Primer sequence	T_m_ (°C)	Location
1	*Pm05_707*	Forward^b^	GGTAGAAGGAGCAACGGACA	63.5	Chromosome 5
		Reverse	CGCTCGGGTCATCGTTATTA	61.3	
2	*Pm06_506*	Forward^b^	TTGTGCGTATGCAACCTTTC	57.6	Chromosome 6
		Reverse	CAAAAGGGAAGGAGCACAAA	57.0	
3	*Pm07_429*	Forward^b^	TTCCTTTTCATCCTCTGCAA	59.1	Chromosome 7
		Reverse	CGAATGAGAGTAGTGCGGAAA	62.8	
4	*Pm09_801*	Forward^b^	TGACTTCGGTTAGAATATGTTTGC	60.7	Chromosome 9
		Reverse	TCACACTCCTTTCAATTTCTCA	59.4	
5	*Pm12_426*	Forward^b^	TTCGTGTTCTCGCTTTCCTC	62.0	Chromosome 12
		Reverse	GATCACTTCGCACGGGATAG	61.9	
6	*Pm13_110*	Forward^b^	TCAAGTGGAATAACCGCAAG	60.0	Chromosome 13
		Reverse	CAGACGAGGACTTTCATTTCG	60.4	

^a^Novel microsatellite markers for *Plasmodium malariae* based on fragment analysis of PCR products (N = 37)

^b^Forward primers were labelled with 6-FAM for fragment analysis. Thermal cyclin profile: initial denaturation at 94 °C for 5 min, 40 cycles of 94 °C for 1 min, 58–63 °C for 1 min and 72 °C for 1 min, followed by a final extension at 72 °C for 5 min

**Table 2:** Primary and secondary primers used for amplification of *pmmsp1* gene located on chromosome seven using semi-nested PCR

S. no.	Locus	Primer	Primer sequence	T_m_ (°C)
1	Primary^a^	PMMSP1full_F2 (N1F)	GAATTGTCGAAAGCATTGGT	54.2
		PMMSP1full_OR2 (N1R)	TCAACTTCTTTCTTTTCTGCTTC	55.0
2	Secondary^b^	PMMSP1VNTR_1F (NF2)	CCAAGCATACGGAACAGGAG	58.8
		PMMSP1VNTR_1R (NR2)	CAAATCTAATTGGTCGCACTTC	56.2

Thermal cycling profile: initial denaturation step at 95 °C for 5 min, followed by 25 cycles of denaturation at 94 °C for 1 min, annealing at 55 °C for 2 min and extension at 72 °C for 2 min then last extension step at 72 °C for 5 min. 2 µL of each primary reaction was used as template for the 100 µL secondary PCR reaction. Thermal cycling profile: Initial denaturation step at 95 °C for 5 min, followed by 30 cycles of denaturation at 94 °C for 1 min, annealing at 58 °C for 2 min and extension at 72 °C for 2 min then final extension step at 72 °C for 5 min

^a^Primary and ^b^Secondary set of primers were used to amplify the *pmmsp1* gene segment of *Plasmodium malariae*

### Validation of markers using PCR analysis

Primers for microsatellite genotyping markers were labelled with 6-Carboxyfluorescein (6-FAM) and validated using PCR (Table 1) followed by fragment analysis. PCR were performed in Eppendorf Mastercycler^®^ pro (Eppendorf, Germany) with a total volume of 20 μL containing 1× PCR Buffer II (Mg^2+^ free), 2–3 mM MgCl_2_, 125 μM dNTPs, 0.25 μM primers and 0.4 U/20 μL of Platinum Taq Polymerase (Invitrogen, USA). Gel electrophoresis was used to detect the amplified products on 3% agarose gel. Fragment analysis of the 6-FAM-labelled PCR products was conducted using gel capillary electrophoresis by Macrogen (Macrogen Inc., South Korea). During fragment analysis, presence of a distinct expected single peak with a minimum of 100 relative fluorescent units (RFU) were accepted as cut-off value. If multiple peaks were detected, then one-third height of dominant peak with minimum of corresponding proportionate RFUs were taken as selection threshold for scoring the multiple recessive microsatellite alleles per locus.

For *pmmsp1* polymorphisms, semi-nested PCR reaction was conducted (Table 2) to increase sensitivity. Primary and secondary PCR products were generated using corresponding volumes of 20 and 100 µL reaction in the presence of 10 mM Tris–HCl, pH 8.3, 50 mM KCl, 2 mM MgCl_2_, 125 µM dNTPs, 125 nM of each primers and 0.4 units of Platinum Taq Polymerase (Table 2). Sequences of amplified products were obtained using high-fidelity capillary electrophoresis conducted by Macrogen. Mono infection of *P. malariae* DNA verified using 18S rRNA-based PCR was taken as positive control. The PCR master mix with nuclease free water instead of parasite DNA was taken as control.

### Multiplicity of infections

As the blood-stage malaria parasites are haploid, the presence of multiple peaks during evaluation of fragment size or VNTR analysis for one or more alleles at target locus was inferred as co-infection with two or more genetically distinct variants. This was referred to as multiplicity of infections (MOI) for the same isolate [20, 21, 67]. The mean MOI for positive samples was calculated independently for each marker by dividing the total number of *P. malariae* clones identified by the number of samples PCR positive for the parasite. For microsatellites, the single or predominant locus at each allele was considered for evaluating allele frequencies. The allele fragment size was interpreted using GeneScan™ version 3.1. Additional alleles were scored only if the peak height was at least one-third the height of the major peak during fragment analysis. For *pmmsp1* gene, sequence data were interpreted using Bioedit version 7.0.4. Allelic frequencies for *pmmsp1* gene were determined based on length of consensus sequences for variable number of tandem repeats (VNTR) in semi-conserved regions interspersed between coding blocks (Fig. 1).

### Measures of diversity

The expected heterozygosity (*H*_*E*_) was calculated using FSTAT version 1.2 based on previously described formula ${\text{H}}_{\text{E}} = \left[ {{{\text{n}} \mathord{\left/ {\vphantom {{\text{n}} {{\text{n}} - 1}}} \right. \kern-0pt} {{\text{n}} - 1}}} \right]\left[ {1 - \sum\nolimits_{i}^{n} { ={_1}p^{2}} } \right]$ where *p* is the frequency of the *i*th allele and n is the number of alleles sampled [26]. LIAN version 3.7 was used for analyzing overall multilocus linkage disequilibrium (*LD*) implementing a standardized index of association ($I_{A}^{S}$). LD for candidate genotyping markers with *p*-values < 0.05 was considered as significant [68]. Dendogram for microsatellite fragment analysis data was constructed using ClustVis [69]. Blastx (https://blast.ncbi.nlm.nih.gov/Blast.cgi?LINK_LOC=blasthome&PAGE_TYPE=BlastSearch&PROGRAM=blastx) without low-complexity filter was used for identification of regions targeted by primers. Tandemly repeated sequences and copy numbers were identified by using TRF version 4.09 [70]. The number of haplotypes (H), haplotype diversity (H_d_) and pairwise nucleotide diversity (π) were evaluated using DnaSP v5 [34]. Phylogenetic tree was constructed using Mega version 7.0 [71].

### Likelihood of coinfections using genetic markers

The likelihood of two infections by same genotype were deduced by combining individual probabilities of two or more unlinked genetic markers and designated as combined probabilities (πP_i_), where πP_i_ = P_1_ × P_2_ × ⋯P_i_, where $\text{P}_{i} = \sum {p_{i}^{2} }$ is individual probabilities for each markers being utilized. The assumption was based on each infections being independent and the probability of reinfection by same genotype were products of probabilities for individual markers [72].

### Sensitivity and specificity

The specificity of primers was assessed on samples from symptomatic *P. malariae* patients (Additional file 1: Table S2) compared to the reference nested-PCR method targeting the 18S rRNA [60]. Specificity of all primer products were checked for amplification of unspecific products to access true positive results. The specificity of the markers was tested by two approaches: (i) BLAST analysis of the primers sequences against the NCBI databases and in silico PCR using default settings of UGENE version 1.30 [73] against whole genome reference database [64] of all available human infecting *Plasmodium* species. All markers did not belong or cross-aligned with any regions of other *Plasmodium* species. (ii) The samples used were verified for mono-infection of *P. malariae* pre-confirmed using 18S rRNA-based PCR specific. During PCR validation, all markers were specific to *P. malariae* only and had no cross reactivity to *P. falciparum* and *P. vivax*. (iii) Screening for sequence polymorphisms in the *pmmsp1* gene by comparing to reference sequence or fragments analysis of amplified products. Non-specific amplification was not observed for any markers during assessment of qPCR products by gel electrophoresis. For microsatellite markers, inspection of potential contamination was safeguarded by routine insertion of known negative samples in each PCR run to access true negative results. For all six microsatellite markers except one sample for Pm06_506 (1/37), the target amplification was positive for all symptomatic *P. malariae* patients (N = 37), thus making sensitivity and specificity ~ 100% (Additional file 1: Table S3). For primers targeting the *pmmsp1* gene polymorphisms, all well-sequenced samples (N = 27) exhibited VNTR polymorphisms and non-specific amplification or false positive results were not identified (Additional file 1: Table S3). Additional inspection of potential contamination involved cross-comparison of the amplified region with known sequences from *P. falciparum* and *P. vivax*. To avoid cross-contamination of samples during DNA addition and PCR processing steps, negative controls consisting of only water were added in each run.

## Results

### Identification of novel markers

**Fig. 2:**
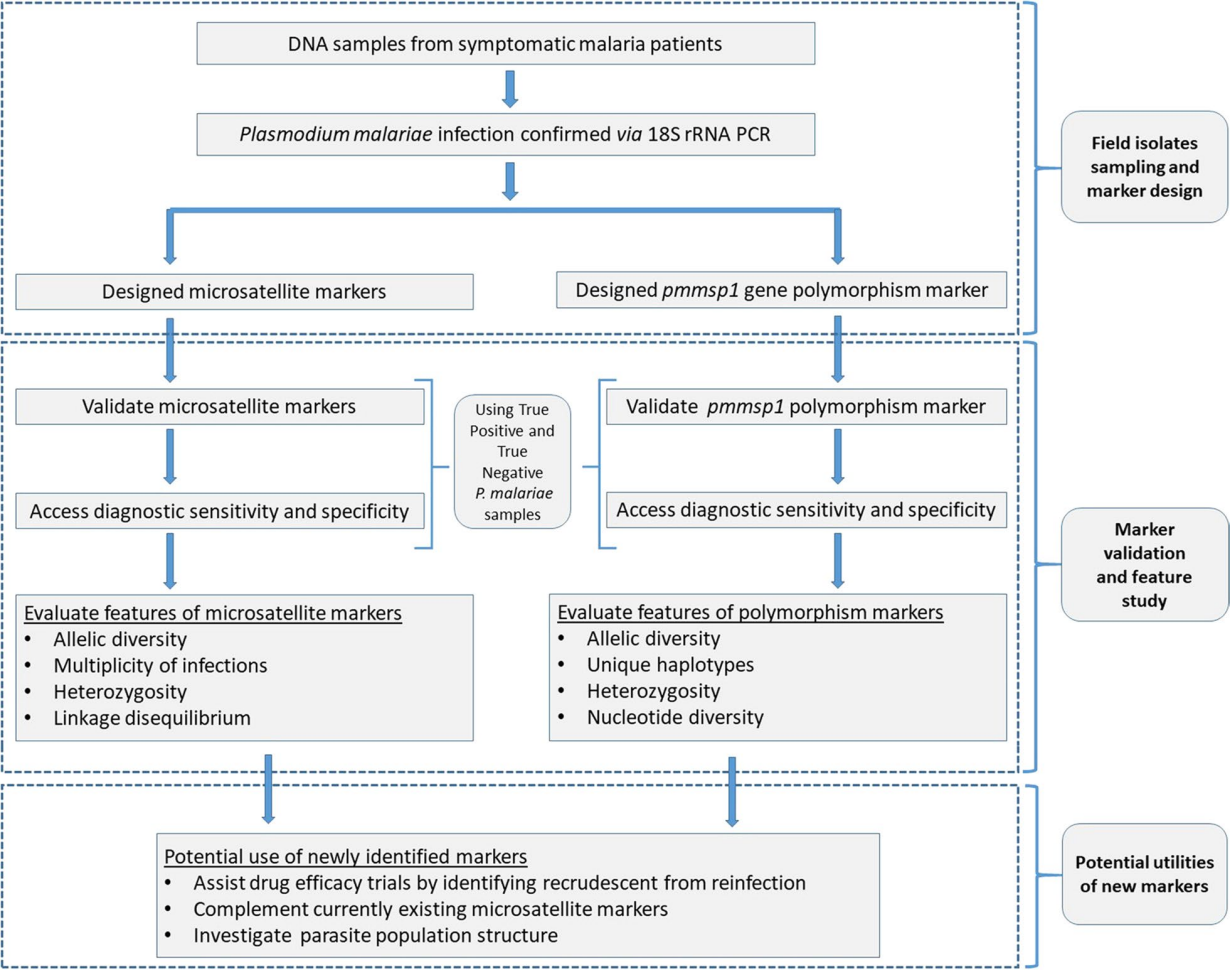
Schematic overview of the *Plasmodium malariae* molecular marker development and validation used in the current study and their potential implementation

Figure 2 summarizes the approach we used to develop and validate genetic markers in *P. malariae*. To determine suitable *P. malariae* genotyping markers, the prevalence of polymorphisms for potential markers in 37 symptomatic *P. malariae*-positive blood samples from Myanmar was evaluated (Additional file 1: Table S2). Six promising genotyping markers, namely, Pm05_707, Pm06_506, Pm07_429, Pm09_801, Pm12_426 and Pm13_110 (Table 1) were identified. For all markers polymorphic fragments could be generated in most *P. malariae*-positive test samples (Additional file 1: Table S3). Blastx analysis showed that the regions covered by these markers were not linked to known CDS or experimentally verified protein expressing regions of the parasite genome. For the *pmmsp1* gene, a gene sequence could be generated in 27 out of the 37 parasite samples, in which VNTR-related INDEL polymorphisms could be evaluated. The majority of the VNTRs belonged to a semi-conserved region in block 8 of *pmmsp1* gene (Fig. 1) and contained imperfect repeats of the consensus sequence with a length of 27 bp and with copy numbers in range 2.4–23.9 resulting in allele length in range 64–645 bp (Additional file 2: Supplementary information). In the remaining samples, lack of PCR efficiency was the most likely cause of failure to amplify the *pmmsp1* gene.

**Fig. 3:**
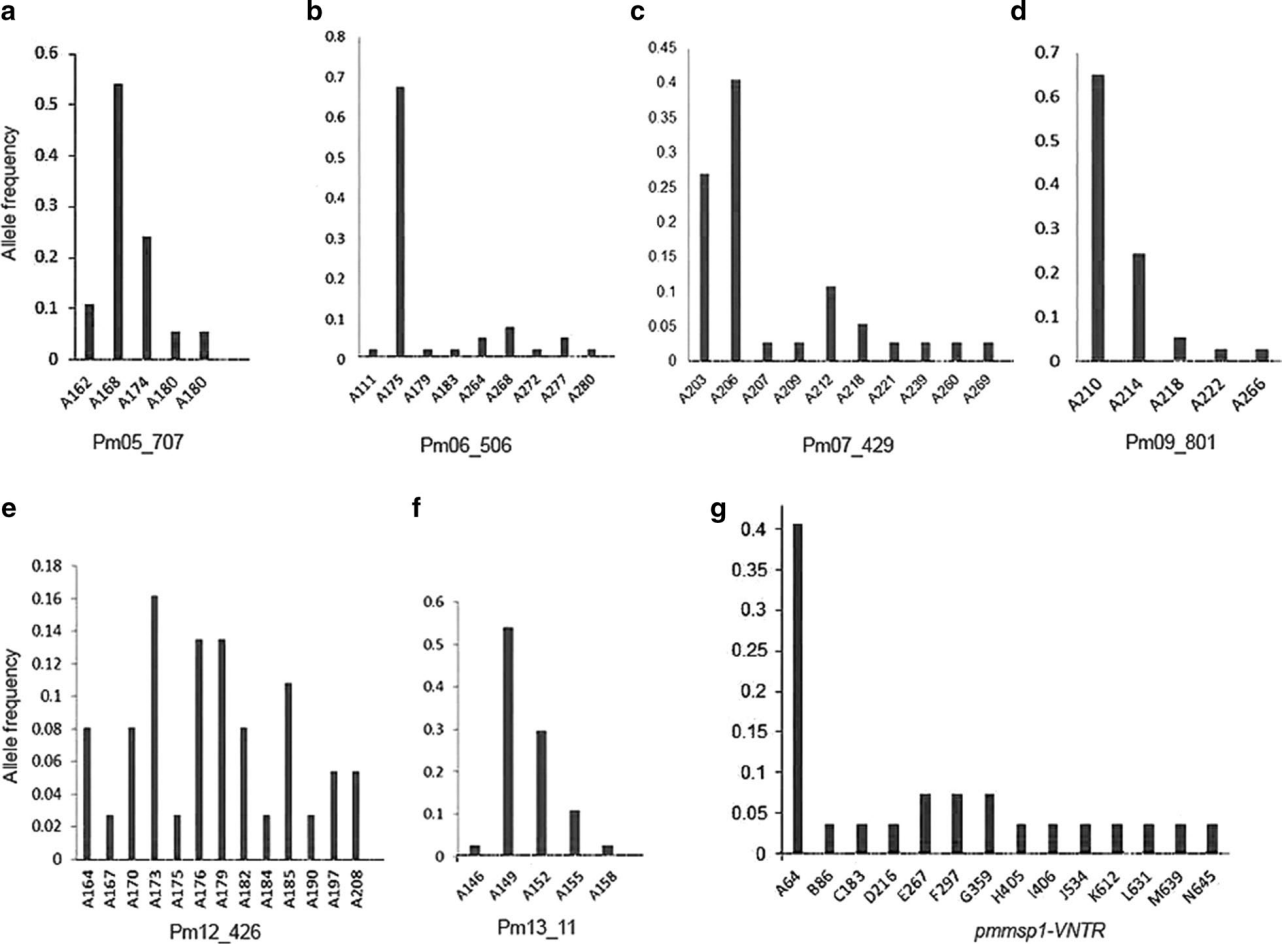
Genetic richness and allele frequency. Allele frequencies observed for each microsatellite markers (**a**) Pm05_707, (**b**) Pm06_506, (**c**) Pm07_429, (**d**) Pm09_801, (**e**) Pm12_426 and (**f**) Pm13_11, respectively. **g** Allele frequency based on VNTR observed for *P. malariae msp1* gene. VNTR, variable number of tandem repeats; *msp,* merozoite surface protein

### Allelic frequencies and measures of marker diversity

**Fig. 4:**
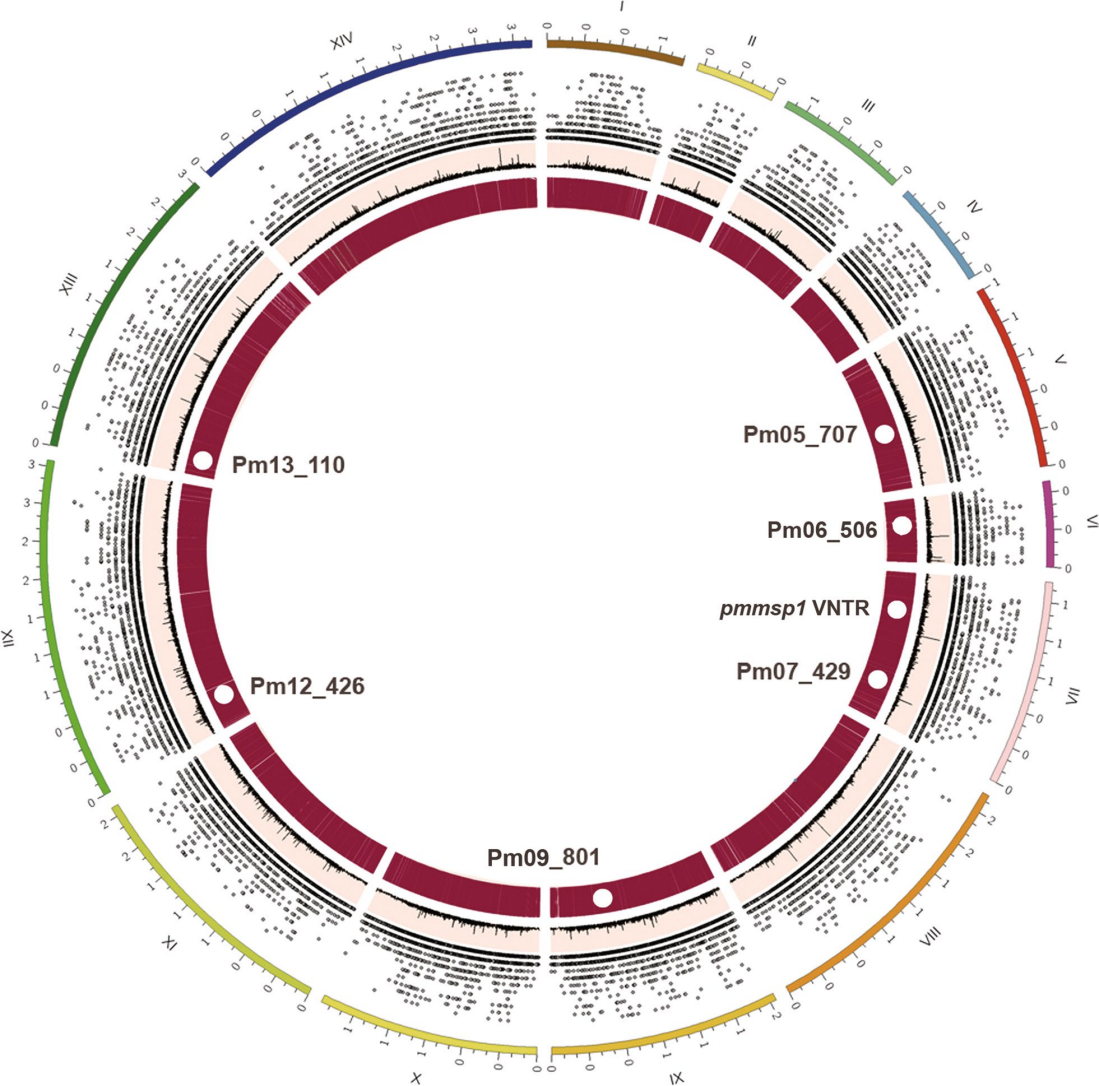
Location of the genotyping markers in *Plasmodium malariae* UG01. Different features indicated by the map for microsatellite distribution from outermost to innermost ring can be interpreted as: chromosome 1-14 (I-XIV) in Mbp, scatter plot^a^ for genomic microsatellite distribution based on unit motif length which corresponds to the height of spot from base of its ring, line plot with peaks indicating regions with long repeat length and heatmap^b^ corresponding to the aggregate genomic microsatellite for the region. ^a^Spots and regions in scatter plot and ^b^heatmap may appear overlapped due to high density but are physically apart in sequence. The approximate location of newly identified genotyping markers are indicated with white circles and labeled accordingly; Mbs, mega base pairs

The six microsatellite markers, namely, Pm05_707, Pm06_506, Pm07_429, Pm09_801, Pm12_426 and Pm13_110 displayed corresponding *H*_*E*_ (distinct alleles) of 0.649(5), 0.542(9), 0.764(10), 0.530(5), 0.922(13) and 0.623(5), respectively. Observed allele size ranged from 146 to 269 bp resulting in high diversity of allele frequencies (Fig. 3a–f). Candidate genotyping markers were situated on different chromosomes (Table 1, Fig. 4). Linkage analysis showed a *I*_*A*_^*S*^ = 0.03 (*p*-*value* > 0.05), suggesting absence of a linkage disequilibrium. Sequence analysis of VNTR-based allelic frequencies in semi-conserved regions of *pmmsp1* gene interspersed between coding blocks identified 14 distinct alleles (Fig. 3g). A copy number of 2.4 with consensus sequence “GAACAAGCAGAAACAACGGGAACAACA” located at *pmmsp1* nucleotide position 2312–2374 bp was the most frequent observed VNTR (40%). Considerable nucleotide diversity (π = 0.0976) and haplotype gene diversity (*H*_*d*_ = 0.698) were observed. Linkage disequilibrium was significant (*p*-*value *< 0.01) for segregating sites. The combined likelihood that any two samples by chance would have the same genotypes was as low as 8.46 × 10^−4^ for six microsatellites markers and further reduced to 1.68 × 10^−4^ when *pmmsp1* polymorphisms were included (Table 3).

**Table 3:** Probabilities of the coincidence of the same *Plasmodium malariae* genotype

S. no.	Molecular marker^a^	Probability (${\text{P}} = \sum {p_{i}^{2} }$) for each marker	Combined probabilities	Combined probability values (πP_i_)^b^
1	Pm12_426	0.102	P1 = P_Pm12_426_	1.02000 ×10^−1^
2	Pm07_429	0.255	P2 = P2 × P_Pm07_429_	2.60100 ×10^−2^
3	Pm05_707	0.369	P3 = P3 × P_Pm05_707_	9.59769 ×10^−3^
4	Pm13_110	0.394	P4 = P4 × P_Pm13_110_	3.78149 ×10^−3^
5	Pm06_506	0.462	P5 = P5 × P_Pm06_506_	1.74705 ×10^−3^
6	Pm09_801	0.484	P6 = P6 × P_Pm09_801_	8.45571 ×10^−4^
7	*pmmsp1*	0.196	P7 = P7 × P_*pmmsp1*_	1.65732 ×10^−4^

^a^Ordered according to increasing power of P for microsatellite genotyping markers except for *pmmsp1* due to difference in marker type

^b^Combined cumulative probabilities πP_i_ calculated as πP_i_ = P_1_ × P_2_ × …P_i_

### Identification of MOI by genetic markers

Genotyping markers identified up to 13 distinct alleles for microsatellite markers and 14 for *pmmsp1* markers (Fig. 3). Pm12_426 displayed the highest mean MOI (1.216) followed by Pm05_707 (Table 4). Because of their high allelic diversity and heterozygosity, the Pm12_426 and *pmmsp1* markers were most potent for detecting MOI (Fig. 3, Table 3).

**Table 4:** Characteristics of polymorphic microsatellite loci detected in *Plasmodium malariae* (N = 37)

S. no.	Locus	Repeat unit^a^	Allele size range (bp)	Total no. of alleles detected^b^	No. of distinct alleles	Expected heterozygosity (H_E_)^c^	Mean MOI
1	Pm05_707	(AAT)^9^	162–180	42	5	0.649	1.135
2	Pm06_506	(ACAT)^40^	175–280	41	9	0.542	1.081
3	Pm07_429	(AAT)^14^	203–269	40	10	0.764	1.081
5	Pm09_801	(ACAT)^9^	210–266	40	5	0.530	1.081
4	Pm12_426	(ATC)^21^	164–208	45	13	0.922	1.216
6	Pm13_110	(AAT)^10^	146–158	39	5	0.623	1.054
7	*pmmsp1*	VNTR^d^	64–645	27	14	0.835	1.000

^a^The repeat number of each microsatellite unit motif

^b^Total numbers of alleles including both dominant and minor alleles detected

^c^Expected Heterozygosity (H_E_) was calculated from a restricted data set containing only the dominant allele in each sample

^d^The allele based on consensus sequence of variable number of tandem repeats

## Discussion

The genetic epidemiology of *P. malaria* is largely unknown. Availability of a comprehensive set of appropriate genetic markers is a prerequisite for advancing this field. The present study identifies six genotyping microsatellites and a set of VNTR-based *pmmsp1* INDEL polymorphism markers in *P. malariae*. In contrast, allelic frequencies of microsatellites are generally higher and more evenly distributed due to absence of selective pressure. The newly identified six genotyping markers for *P. malariae*, supplement the widely used microsatellite markers developed for *P. falciparum* [21, 28] and *P. vivax* [20, 74]. Unlike some of the previous markers [13], all presently identified markers were located in different chromosomes and did not show any significant linkage disequilibrium, suggesting better discriminative potentials of these markers. Microsatellite markers are valuable tools for multilocus genotyping, and newly identified markers were able to clearly discriminate multiple *P. malariae* genotypes in naturally acquired symptomatic infections. Comparison of discriminative power associated with different genotyping markers in separate studies is complicated and often incomparable since heterozygosity values for a single marker can greatly vary between study sites [20, 72]. The choice of marker, genotyping technique and underlying genetic diversity of study groups affect outcomes of each study. Nonetheless, the heterozygosity displayed by six newly identified (0.542–0.922) and six previously reported (0.192–0.849) microsatellite markers for *P. malariae* [13, 14] could be used together to access higher polymorphisms with samples involving geographically large regions. Moreover, the combined set of markers would facilitate microsatellite genotype of *P. malariae* populations in similar ranks to those of *P. vivax* [67, 75] and *P. falciparum* [28, 76] which generally involves use of 10–13 sets of microsatellite markers. In particular, the newly identified marker Pm12_426 expressed *H*_*E*_ value of 0.922 which was noticeably higher than previously reported highest *H*_*E*_ of 0.811 [13]. The observed *H*_*E*_ are promising, since the samples involved for validation were collected within same year and from geographically small region. Such features with high heterozygosity tend to make these markers suitable candidate for linkage mapping, which requires highly polymorphic markers [77]. The mean MOI for newly identified microsatellite markers (1.2 ± 0.1), was appreciable compared to previously published markers with mean MOI 1.12–1.32 [13]. Observed allelic diversity, high sensitivity and specificity (≥ 97%) for all six microsatellite markers suggests promising potency for population structure and epidemiological studies. All markers were unlikely to be in coding regions and were positioned within highly repetitive and AT-rich regions of the genome, which increases likelihood of these markers achieving higher heterozygosity in larger population size [78]. Likewise, the combined likelihood enhances the sensitivity of these markers by highly reducing identification of same genotype by chance, which enables utility of the markers in low transmission settings. The microsatellite genotyping method is relatively inexpensive compared to INDEL and SNP analysis [79, 80]. Moreover, the amplification and genotyping stages might be adopted to multiplex different loci, saving costs, time and facilitating large scale studies [23, 25].

VNTR-targeted INDEL analysis indicated promising ability of *pmmsp1* gene polymorphisms to identify variants within the study population. The most frequently identified VNTRs with high heterozygosity were situated in *pmmsp1* semi-conserved variable block 8, making it a potential genetic marker for *P. malariae* population study. The *msp1* gene block 8 in previous study for *P. falciparum* and *P. vivax* had less than 45% interspecies identity 1 [40]. Presence of polymorphisms for *msp1* block-2 have been reported for *P. falciparum* [81, 82], however, information on the *P. malariae* homologue is scarce. In present study, the *pmmsp1* polymorphisms marker showed highly imbalanced distribution of its most common allele (40% for the A64 fragment) which might be explained by natural selection. The observed linkage disequilibrium for *pmmsp1* polymorphisms is likely caused by SNPs grouped into haplotype blocks which often harbour limited number of distinct haplotypes [83]. Unlike microsatellites, the larger differences arising due to variation in copy numbers from VNTR in the amplified region is easily visualized by gel electrophoresis facilitating easy interpretation. However, template DNA required for INDEL analysis was more vulnerable to low DNA template quality which likely related to the larger amplicon size, and resulted in a smaller number of sample with successful amplification of the *pmmsp1* gene. Nonetheless, the high allelic diversity and heterozygosity observed for well-sequenced samples indicated feasibility of this polymorphisms to be exploited for population genetic studies. The mean MOI for newly identified microsatellite markers were in range 1.10–1.20, similar to previously reported markers with mean MOI range of 1.12–1.32 [13]. The differences in observed genotypes and MOI by Pm12_426 and *pmmsp1* markers for same population could indicate either greater transmission intensities or merely differences in the resolution of these molecular markers. Results from dendrograms and phylogenetic tree suggested similar outcomes while utilizing these markers to cluster population (Additional file 3: Fig. S1) and identify highly segregating variants.

## Conclusions

In summary, the newly developed genotyping microsatellite markers and *pmmsp1* gene polymorphisms may provide an important tool for studies in *P. malariae.* Practical applications include discrimination between disease recrudescence and reinfection in drug efficacy trials, studies on gene flow, parasite selection, population relatedness, signatures of selection and genetic diversity as a measure of transmission intensity and other genetic epidemiological questions. In addition to microsatellite typing, the VNTR-associated polymorphisms observed in semi-conserved block 8 of *pmmsp1* gene are useful for assessing genetic diversity in *P. malariae*.

## Supplementary information

**Additional file 1: Table S1.** Genome-wide coverage and density of microsatellites in genome of five human malaria causing *Plasmodium* species. **Table S2.** Number and geographical origin of *Plasmodium* species and strains used in the present study. **Table S3.** Sensitivity and specificity estimates of the markers in method development.

**Additional file 2.** Schematic diagram of partial *pmmsp1* gene amplification and VNTR alleles alignment. The diagram is representative of 14 alleles aligned to the partially sequenced *pmmsp1* gene reference sequence (Accession no. FJ824669) using *pmmsp1* marker described in Table 2. VNTR, variable number of tandem repeats; *pmmsp1, Plasmodium malariae merozoite surface protein 1.*

**Additional file 3: Figure S1.** Dendrogram and phylogenetic tree for *P. malariae* samples (A) Dendrogram was constructed for microsatellite markers with rows clustered using correlation distance and complete linkage. The columns were clustered using correlation distance and average linkage (N = 37). (B) Phylogenetic tree constructed ws constructed using neighbor joining method for *pmmsp1* gene sequence INDEL polymorphisms (N = 27). Figure A and B are not equivalent representative of the population clustering as type of genetic markers and sample size used for analysis are different.

## Data Availability

The dataset generated during the current study are available from corresponding author on reasonable request.

## Abbreviations

bp: base pair
CDS: protein-coding-regions
*H*_*E*_: heterozygosity
*H*_*d*_: genetic diversity
INDEL: insertion deletion
Kbp: kilo base pair
Mbp: million base pair
*msp*: merozoite surface protein
ORFs: open reading frame
RFU: relative fluorescent unit
SNP: single nucleotide polymorphisms
VNTR: variable number tandem repeat
