# Supplementary material for: Polymorphic markers for identification of parasite population in Plasmodium malariae
Source: Malar J. 2020 Jan 28;19:48. doi: 10.1186/s12936-020-3122-2 (PMC6988369; doi:10.1186/s12936-020-3122-2)
Supplement: Supplementary file 1 — Additional file 1: Table S1. Genome-wide coverage and density of microsatellites in genome of five human malaria causing Plasmodium species. Table S2. Number and geographical origin of Plasmodium species and strains used in the present study. Table S3. Sensitivity and specificity estimates of the markers in method development. [file 12936_2020_3122_MOESM1_ESM.docx]

**Additional file 1**

**Table S1.** Genome-wide coverage and density of microsatellites in genome of five human malaria causing *Plasmodium* species

| **Imperfect repeat features** | **P. falciparum 3D7** | **P. vivax**  **SAL-1** | **P. malariae**  **UG01** | **P. ovale curtisi**  **GH01** | **P. knowlesi STRAIN-H** |
| --- | --- | --- | --- | --- | --- |
| Sequence analyzed (Mbp) | 23.33 | 27.01 | 33.61 | 33.47 | 24.39 |
| Genomic GC  content (%) | 19.34 | 42.19 | 24.38 | 28.48 | 38.60 |
| No. of microsatellite | 84786 | 20875 | 47762 | 29245 | 32008 |
| Density of microsatellite (No./Mbp) | 3633 | 772 | 1420 | 873 | 1312 |
| Occurrence of microsatellite per 2 Kb | 7.3 | 1.545 | 2.84 | 1.747 | 2.624 |
| Total length of microsatellite (kbp) | 2698 | 591 | 1663 | 793 | 1129 |
| microsatellite Coverage (bp/Mbp) | 115634 | 21878 | 49471 | 23692 | 46285 |
| Percentage of perfect microsatellites (%) | 56.09 | 70.22 | 61.71 | 76.70 | 71.32 |
| Genome content by microsatellite (%) | 11.56 | 2.19 | 4.95 | 2.37 | 4.63 |

**Table S2.** Number and geographical origin of *Plasmodium* species and strains used in the present study

| **S.N.** | **Plasmodium species ^a^** | **n** |
| --- | --- | --- |
| 1 | *P. malariae* mono-infection | 31 |
| 2 | *P. malariae + P. falciparum* | 4 |
| 3 | *P. malariae + P. vivax* | 2 |
|  | Total samples (N) | 37 |

^a^results are based on identification of parasite present in samples using 18sRNA PCR [60]

**Table S3.** Sensitivity and specificity estimates of the markers in method development

| **results** | **known test samples** | | | | | | | | | | | | | |
| --- | --- | --- | --- | --- | --- | --- | --- | --- | --- | --- | --- | --- | --- | --- |
|  | Pm05_707 | | Pm06_506 | | Pm07_429 | | Pm09_801 | | Pm12_426 | | Pm13_110 | | *pmmsp1* VNTR | |
|  | positive | negative | positive | negative | positive | negative | positive | negative | positive | negative | positive | negative | positive | negative |
| positive | ^37^ | ^0^ | ^36^ | ^0^ | ^37^ | ^0^ | ^37^ | ^0^ | ^37^ | ^0^ | ^37^ | ^0^ | ^27^ | ^0^ |
| negative | ^0^ | ^2^ | ^0^ | ^5^ | ^0^ | ^2^ | ^0^ | ^2^ | ^0^ | ^2^ | ^0^ | ^2^ | ^0^ | ^2^ |

^a^Sensitivity: true positive (TP)/(TP + false negative [FN]), 100% (37/37+0); diagnostic specificity: TN/(TN + FP), 100% (2/2+0).
