# Supplementary material for: Polymorphic markers for identification of parasite population in Plasmodium malariae
Source: Malar J. 2020 Jan 28;19:48. doi: 10.1186/s12936-020-3122-2 (PMC6988369; doi:10.1186/s12936-020-3122-2)
Supplement: Supplementary file 2 — Additional file 2. Schematic diagram of partial pmmsp1 gene amplification and VNTR alleles alignment. The diagram is representative of 14 alleles aligned to the partially sequenced pmmsp1 gene reference sequence (Accession no. FJ824669) using pmmsp1 marker described in Table 2. VNTR, variable number of tandem repeats; pmmsp1, Plasmodium malariae merozoite surface protein 1. [file 12936_2020_3122_MOESM2_ESM.pdf]

Additional file 2: Alignment of 14 *pmmsp1* alleles aligned to *pmmsp1* reference gene (Accession no. FJ824669)

```
                             10        20        30        40        50        60
                    ....|....|....|....|....|....|....|....|....|....|....|....|
PMMSP1(FJ824669)    AAAAAATGAGAAAAAACTGGAGGAAGAAAAAATCAAGGCGGCAGCACAAAATGTAAGCGG
A64_PMMSP1          ............................................................
B86_PMMSP1          ............................................................
C183_PMMSP1         ............................................................
D216_PMMSP1         ............................................................
E267_PMMSP1         ............................................................
F297_PMMSP1         ............................................................
G359_PMMSP1         ............................................................
H405_PMMSP1         ............................................................
I406_PMMSP1         ............................................................
J534_PMMSP1         ............................................................
K612_PMMSP1         ............................................................
L631_PMMSP1         ............................................................
M639_PMMSP1         ............................................................
N645_PMMSP1         ............................................................

                             70        80        90       100       110       120
                    ....|....|....|....|....|....|....|....|....|....|....|....|
PMMSP1(FJ824669)    CCATGTTTCCGGAGCAGATGAAACATCAAACAGTCATGGATCATCTGGAGGAGGAGGATC
A64_PMMSP1          ............................................................
B86_PMMSP1          .........................C.........C..............C.AC....A.
C183_PMMSP1         ............................................................
D216_PMMSP1         ............................................................
E267_PMMSP1         ............................................................
F297_PMMSP1         ............................................................
G359_PMMSP1         ............................................................
H405_PMMSP1         .........................C........TC..............C.AC....A.
I406_PMMSP1         .........................C.........C..............C.AC....A.
J534_PMMSP1         .........................C........TC..............C.AC....A.
K612_PMMSP1         .........................C........TC..............C.AC....A.
L631_PMMSP1         ............................................................
M639_PMMSP1         ............................................................
N645_PMMSP1         .........................C........TC..............C.AC....A.

                            130       140       150       160       170       180
                    ....|....|....|....|....|....|....|....|....|....|....|....|
PMMSP1(FJ824669)    AACACAAACAGTAACGACAACACCATCAACTACAACAACAGCAACTACATCATCACAAAC
A64_PMMSP1          ............................................................
B86_PMMSP1          ....GG.CA..C-------------------.GG....--...GGACA.G..GG.GC...
C183_PMMSP1         .....G.........A............................................
D216_PMMSP1         ............................................................
E267_PMMSP1         .....G......................................................
F297_PMMSP1         ............................................................
G359_PMMSP1         ............................................................
H405_PMMSP1         ....GG.CA..CTGGAG.....GG.A....-.GG....--...GGACA.G..GG....G.
I406_PMMSP1         ....GG.CA..CTGGAG.....GG.A....-.GG....--..TGGA--------------
J534_PMMSP1         ....GG.CA..CTGGAG.....GG.A....-.GG....--...GGACA.G..GG....G.
K612_PMMSP1         ....GG.CA..CTGGAG.....GG.A....-.GG....--...GGACA.G..GG....G.
L631_PMMSP1         ............................................................
M639_PMMSP1         .....G.......G.A............................................
N645_PMMSP1         ....GG.CA..CTGGAG.....GG.A....-.GG....--...GGACA.G..GG....G.

                            190       200       210       220       230       240
                    ....|....|....|....|....|....|....|....|....|....|....|....|
PMMSP1(FJ824669)    AGTATCAGTAGGAGAAACAGGATCAGCACAAGCA-CAAGCACAAC--CACAACCACAACC
A64_PMMSP1          ..................................-.........G--.............
B86_PMMSP1          G.G.A..AC....C..G.....CA...TGG....A..GAACA.G.AAG....G.TGG.G.
C183_PMMSP1         ..................................-..........--.............
D216_PMMSP1         ..................................-.........G--.....G.......
E267_PMMSP1         ..................................-.........G--.............
F297_PMMSP1         ..................................-.........G--.............
G359_PMMSP1         ..................................-..........--.............
H405_PMMSP1         T.G.G..ACG...AC.......CA...GG....A.GG.A.....AGG....G..GG.G.
I406_PMMSP1         ------------------------------------------------------------
J534_PMMSP1         T.G.G..ACG...AC.......CA...GG....A.GG.A.....AGG....G..GG.G.
K612_PMMSP1         T.G.G..ACG...AC.......CA...GG....A.GG.A.....AGG....G..GG.G.
L631_PMMSP1         ..G.....C.CA..C.CA..C.CAG.T....T..-........G--GT....T.....G.
M639_PMMSP1         GA................................-........G--GT....T.....G.
N645_PMMSP1         T.G.G..ACG...AC.......CA...GG------------------------------

                            250       260       270       280       290       300
                    ....|....|....|....|....|....|....|....|....|....|....|....|
PMMSP1(FJ824669)    A-CAACCACAACC--ACAAAC---ACAAACACAAACACAAACACAACCACAAGCAGCAGG
```

Additional file 2: Alignment of 14 *pmmsp1* alleles aligned to *pmmsp1* reference gene (Accession no. FJ824669)

```
A64_PMMSP1          .-...........--......---....................................
B86_PMMSP1          .A.GGGA.....AGG....G.------------------.GG....G.TGG....A...A
C183_PMMSP1         .-...........--......---..............G...................A.
D216_PMMSP1         .-...........--......---....................................
E267_PMMSP1         .-...........--......---..................................A.
F297_PMMSP1         .-...........--......---....................................
G359_PMMSP1         .-...........--......---....................................
H405_PMMSP1         .A.GGGA.....AGG....G.TGG.GC...GGG....AC.GG....G..GG....A.G..
I406_PMMSP1         ----------------------------------------------------...A....
J534_PMMSP1         .A.GGGA.....AGG....G.TGG.GC....GG....AC.GG....G.TGG....A....
K612_PMMSP1         .A.GGGA.....AGG....G.TGG.GC....GG....AC.GG....G.TGG....A....
L631_PMMSP1         .G..GGAG....AGA....G.------------------.GG....G.TGG....A.G..
M639_PMMSP1         .G..GGAG....AGA....G.------------------.GG....G.TGG....A.G..
N645_PMMSP1         ------------------------.GC...GGG....AC.GG....G..GG....A.G..

                              310       320       330       340       350       360
                    ....|....|....|....|....|....|....|....|....|....|....|....|
PMMSP1(FJ824669)    AGCAACGGGAACACCAGGACAATCAGGACAATCAGGACAATCAGGACAATCAGGAGCTGA
A64_PMMSP1          ................A...........................................
B86_PMMSP1          .CA.G.AA..CA.G.T...GC.A.G...AC.A........G.....GC.A.G...A.AAC
C183_PMMSP1         ................A........A........A..------------------.....
D216_PMMSP1         ..............................................---------.....
E267_PMMSP1         ................A...........................................
F297_PMMSP1         ................A...........................................
G359_PMMSP1         ................A...........................................
H405_PMMSP1         .A....A...CA.G.T...GC.A.....AC.A..--------------------------
I406_PMMSP1         .A....A...CA.G.T...GC.A.....AC.A..--------------------------
J534_PMMSP1         .A....A...CA.G.T...GC.A.....AC.A........G........G.....CAA.C
K612_PMMSP1         .A....A...CA.G.T...GC.A.....AC.A........G........G.....CAA.C
L631_PMMSP1         .A....A...CA.G.....GC.A.G...AC.A........G........G.....CAA.C
M639_PMMSP1         .A....A...CA.G.....GC.A.G...AC.A..------------------...CAA.C
N645_PMMSP1         .A....A...CA.G.T...GC.A.....AC.A..--------------------------

                              370       380       390       400       410       420
                    ....|....|....|....|....|....|....|....|....|....|....|....|
PMMSP1(FJ824669)    AGGAACAACAGAAACTACGGGAACAACAGGACAGGCAGGAACAACGGGAACACCAGAACA
A64_PMMSP1          ............................................................
B86_PMMSP1          ....CA.G.T.G.G.A..A..............A......G....A......A...G...
C183_PMMSP1         ....................................G..................A....
D216_PMMSP1         ............................................................
E267_PMMSP1         ............................................................
F297_PMMSP1         ............................................................
G359_PMMSP1         ............................................................
H405_PMMSP1         -...CA.G.T.G.G.A..A..............A..T...G....A......A..CG...
I406_PMMSP1         -...CA.G.T.G.G.A..A..............A..T...G....A......A-------
J534_PMMSP1         ....G......G...A..A...CA.G.......A......CA.G.T...G..A...G.AC
K612_PMMSP1         T...G......G...A..A...CA.G.......A......CA.G.T...G..A...G.AC
L631_PMMSP1         ....CA.G.T.G.G.A..A.A.CA.G..A....A..T...G...........A...G...
M639_PMMSP1         ....CA.G.T.G.G.A..A.A.CA.G.......A..T...G...........A...G...
N645_PMMSP1         -...CA.G.T.G.G.A..A..............A..T...G....A......A...G...

                              430       440       450       460       470       480
                    ....|....|....|....|....|....|....|....|....|....|....|....|
PMMSP1(FJ824669)    AGCAGCAGCTGCAGGACCA------CAAGCAGAAACTACGGCAACACCAGGACAAGCAGG
A64_PMMSP1          ...................------................G..................
B86_PMMSP1          ...T.G...AA.....A..ACAGGA.......G.G.A....G....A..........T..
C183_PMMSP1         ...................------................G..................
D216_PMMSP1         ...................------................G..................
E267_PMMSP1         ...................------................G..................
F297_PMMSP1         ...................------...........A....G..................
G359_PMMSP1         ...................------................G..................
H405_PMMSP1         .....G.CAA...C..---------......CG...A..ACA.C...TGAC.G.GA.C.A
I406_PMMSP1         --...G.CAA......---------.......G.G.A..A.A.C...A.AC.G.GA.-AA
J534_PMMSP1         .A...G.CAA.......A.GCAGGA.....T.G.G.A....G....A.............
K612_PMMSP1         .A...G.CAA.......A.GCAGGA.....T.G.G.A....G....A.............
L631_PMMSP1         .....G...AA.G...A..ACAGGA.......G.G.A....G....A.............
M639_PMMSP1         .....G...AA.G...A..ACAGGA.......------------------..........
N645_PMMSP1         .....G.CAA......---------.......G.G.A..A.A.C...A.AC.G.GA...A

                              490       500       510       520       530       540
                    ....|....|....|....|....|....|....|....|....|....|....|....|
PMMSP1(FJ824669)    AGCTGCAGGAGCTGCAGGACCACAAGCAGAAACTACGGGAACACCA--GGACAAGCGGGA
A64_PMMSP1          ..............................................--............
B86_PMMSP1          ...AA.....A.AA......---....T.G.G.A..A......A..--........A...
C183_PMMSP1         ..............................................--............
```

Additional file 2: Alignment of 14 *pmmsp1* alleles aligned to *pmmsp1* reference gene (Accession no. FJ824669)

```
D216_PMMSP1         ..............................................--............
E267_PMMSP1         ..............................................--............
F297_PMMSP1         ..............................................--............
G359_PMMSP1         ..............................................--............
H405_PMMSP1         .CTAAA.CA.A.-T..A.-----..ATT.G..TCGTT.ATC.GA..TT.TC...ATT.C.
I406_PMMSP1         ..TA.A..A.A.-T....-----..ATT.G..TCGTT.TTC.GA..TT.TC...ATT.C.
J534_PMMSP1         ...AA.G...A.AA......---....T.G.G.A.........A..--........A...
K612_PMMSP1         ...AA.G...A.AA......---....T.G.G.A.........A..--........A...
L631_PMMSP1         .-.AA--------.......---....T.G.G.A..A.A.CA.G..--A.......T...
M639_PMMSP1         .-.AA--------.......---....T.G.G.A..A.A.CA.G..--........T...
N645_PMMSP1         ..TA.A..A.A.-T....-----..ATT.G.TTCGTT.TTC.GA..TT.TC...ATT.C.

                             550       560       570       580       590       600
                    ....|....|....|....|....|....|....|....|....|....|....|....|
PMMSP1(FJ824669)    GCTGCAGGACCACAAACAGAGACAGAAGTAGAAGAAACT---------------------
A64_PMMSP1          .......................................---------------------
B86_PMMSP1          ..AA.G...A..AC.GG.C.AG.T.G..C.AC..G...AA--------------------
C183_PMMSP1         .......................................---------------------
D216_PMMSP1         .......................................---------------------
E267_PMMSP1         .......................................---------------------
F297_PMMSP1         .......................................---------------------
G359_PMMSP1         .......................................---------------------
H405_PMMSP1         ATAT.TT..A..GCT.T.TGACTTTTT---------------------------------
I406_PMMSP1         ATAT.TT..AA.GCT.T.TGACTTTTT---------------------------------
J534_PMMSP1         ..AA.G...A..-C.GG.C.AG...G..C.ACG.G...A---------------------
K612_PMMSP1         ..AA.G...A..AC.GG.C.AG...G..C.ACG.G...AACAGGACAAGCTGGAAGCAAC
L631_PMMSP1         ..AA.G...A..AC.GG.C.AG...G..C.ACG.G...AA--------------------
M639_PMMSP1         ..AA.G...A..AC.GG.C.AG...G..C.ACG.G...AA--------------------
N645_PMMSP1         ATAT.TT..AA.GCT.T.TGACTTTTT---------------------------------

                             610       620       630       640       650       660
                    ....|....|....|....|....|....|....|....|....|....|....|....|
PMMSP1(FJ824669)    --------CAGGA--AATTGGAATCGTTGTTCCGACATTGTCCAAATTGCAATATCTTGA
A64_PMMSP1          --------.....--.............................................
B86_PMMSP1          --------.....CA.GC....GCAACA.GAA.A...--.GA...GC..G.GC.A.AG..
C183_PMMSP1         --------.....--.............................................
D216_PMMSP1         --------.....--.............................................
E267_PMMSP1         --------.....--.............................................
F297_PMMSP1         --------.....--.............................................
G359_PMMSP1         --------.....--.............................................
H405_PMMSP1         ------------------------------------------------------------
I406_PMMSP1         ------------------------------------------------------------
J534_PMMSP1         --------.....CA.GC....GCA-CA.GAA.A...--.GA..-GC..G.GC.-.AG..
K612_PMMSP1         GGGAACAA.....CA.GCA...GCAACG.GAA.A...--.GA...GC..G.GC.A.AG..
L631_PMMSP1         --------.....CA.GC....GCAACG.AAA.A...--.GA...GC..G.GC.A.AG..
M639_PMMSP1         --------.....CA.GC....GCAACA.GAA.A...--.GA...GC..G.GC.A.AG..
N645_PMMSP1         ------------------------------------------------------------

                             670       680       690       700       710       720
                    ....|....|....|....|....|....|....|....|....|....|....|....|
PMMSP1(FJ824669)    AAAGCTATATGACTTTTT------------------------------------------
A64_PMMSP1          ..................------------------------------------------
B86_PMMSP1          .C.A.AGG.CA.GC.GGAGCAACAGGAACAACAGGACAA---------------------
C183_PMMSP1         ..................GAAAACTGCTTATGTGTGTCATATCAACATTTTGGT------
D216_PMMSP1         ..................------------------------------------------
E267_PMMSP1         ..................------------------------------------------
F297_PMMSP1         ..................------------------------------------------
G359_PMMSP1         ..................------------------------------------------
H405_PMMSP1         ------------------------------------------------------------
I406_PMMSP1         ------------------------------------------------------------
J534_PMMSP1         .C.A.AGG.CA-GC.GGAG-AACAGGAACAACAGGACAG--------------------C
K612_PMMSP1         .C.A.AGG.CA.GC.GGAGCAACAGGAACAACAGGACAAGCTGGAAGCAACAGGAACAAC
L631_PMMSP1         .C.A.AGG.CA.GC.GGAGCAACAGGAACAACAGGACAA---------------------
M639_PMMSP1         .C.A.AGG.CA.GC.GGAGCAACAGGAACAACAGGACAA---------------------
N645_PMMSP1         ------------------------------------------------------------

                             730       740       750       760       770       780
                    ....|....|....|....|....|....|....|....|....|....|....|....|
PMMSP1(FJ824669)    ------------------------------------------------------------
A64_PMMSP1          ------------------------------------------------------------
B86_PMMSP1          -------GCAGGACAAGCAGGA-GCAACAGAACCACAAACAGAGACCGAAGTAGAAGAAA
C183_PMMSP1         -AAATAACTCAACCATGAATGAAACGTTACTACAACAATATAAACTGAAGATAGAAGAAG
D216_PMMSP1         ------------------------------------------------------------
E267_PMMSP1         ------------------------------------------------------------
F297_PMMSP1         ------------------------------------------------------------
```

Additional file 2: Alignment of 14 *pmmsp1* alleles aligned to *pmmsp1* reference gene (Accession no. FJ824669)

```
G359_PMMSP1         ------------------------------------------------------------
H405_PMMSP1         ------------------------------------------------------------
I406_PMMSP1         ------------------------------------------------------------
J534_PMMSP1         AGGACA-GCAGGACA-GCAGGA-GCAACAGAACCACAA-CAGAGACG-AAGTAGAAGAA-
K612_PMMSP1         AGGACAAGCAGGACAAGCAGGA-GCAACAGAACCACAAGCAGAGACGGAAGTAGAAGAAA
L631_PMMSP1         -------GCAGGACAAGCAGGAAGCAACAGAACCACAAACAGAGACCGAAGTAGAAGAAA
M639_PMMSP1         -------GCAGGACAAGCAGGA-GCAACAGAACCACAAGCAGAGACGGAAGTAGAAGAAA
N645_PMMSP1         ------------------------------------------------------------

                            790       800       810       820       830       840
                    ....|....|....|....|....|....|....|....|....|....|....|....|
PMMSP1(FJ824669)    ------------------------------------------------------------
A64_PMMSP1          ------------------------------------------------------------
B86_PMMSP1          CTCAGGAAATTGGAATCGTTGTTCCGACATTGTCCAAATTGCAATATCTTGAAAAGCTAT
C183_PMMSP1         ATAAGAAATTATTAGAGAATGCGCC-----------------------------------
D216_PMMSP1         ------------------------------------------------------------
E267_PMMSP1         ------------------------------------------------------------
F297_PMMSP1         ------------------------------------------------------------
G359_PMMSP1         ------------------------------------------------------------
H405_PMMSP1         ------------------------------------------------------------
I406_PMMSP1         ------------------------------------------------------------
J534_PMMSP1         CTCAGGAATT--GGATCGTTGTTC-GAC-TTGTCCAA-TTGCA-TATCTTGAAA-GCTAT
K612_PMMSP1         CTCAGGAAATTGGAATCGTTGTTCCGACATTGTCCAAATTGCAATATCTTGAAAAGCTAT
L631_PMMSP1         CTCAGGAAATTGGAATCGTTGTTCCGACATTGTCCAAATTGCAATATCTTGAAAAGCTAT
M639_PMMSP1         CTCACGAAATTGGAATCGTTGTTCCGACATTGTCCAAATTGCAGTATCTTGAAAAGCTAT
N645_PMMSP1         ------------------------------------------------------------

                            850
                    ....|....|.
PMMSP1(FJ824669)    -----------
A64_PMMSP1          -----------
B86_PMMSP1          ATGACTTTTT-
C183_PMMSP1         -----------
D216_PMMSP1         -----------
E267_PMMSP1         -----------
F297_PMMSP1         -----------
G359_PMMSP1         -----------
H405_PMMSP1         -----------
I406_PMMSP1         -----------
J534_PMMSP1         ATGACTTTT--
K612_PMMSP1         ATGACTTTTT-
L631_PMMSP1         ATGACTTTTTT
M639_PMMSP1         GTGACTTTTT-
N645_PMMSP1         -----------
```
