# Supplementary figures and images for: Polymorphic markers for identification of parasite population in Plasmodium malariae
Source: Malar J. 2020 Jan 28;19:48. doi: 10.1186/s12936-020-3122-2 (PMC6988369; doi:10.1186/s12936-020-3122-2)

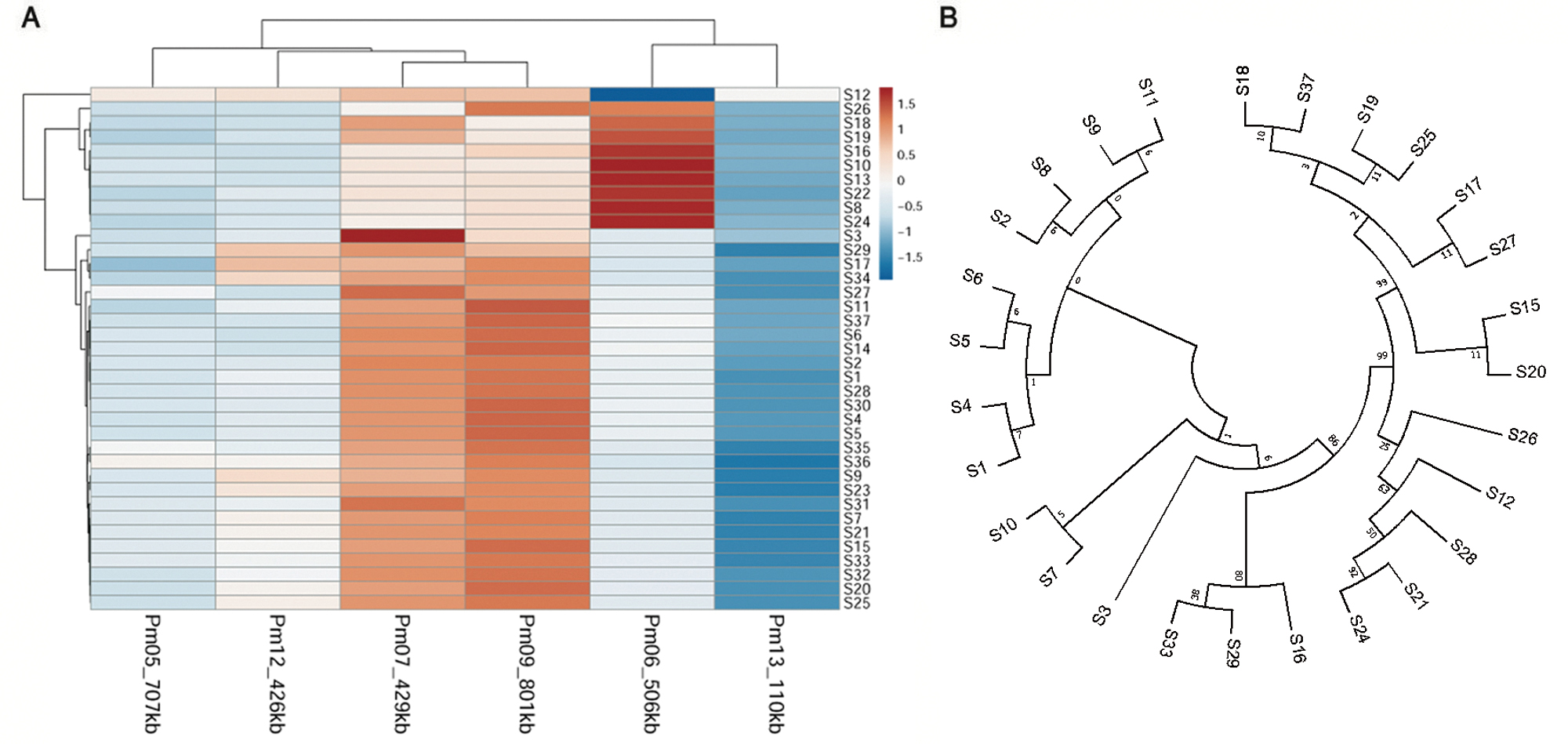

Supplement: Supplementary file 3 — Additional file 3: Figure S1. Dendrogram and phylogenetic tree for P. malariae samples (A) Dendrogram was constructed for microsatellite markers with rows clustered using correlation distance and complete linkage. The columns were clustered using correlation distance and average linkage (N = 37). (B) Phylogenetic tree constructed ws constructed using neighbor joining method for pmmsp1 gene sequence INDEL polymorphisms (N = 27). Figure A and B are not equivalent representative of the population clustering as type of genetic markers and sample size used for analysis are different. [file 12936_2020_3122_MOESM3_ESM.jpg]
